# Auriculotherapy Modulates Macrophage Polarization to Reduce Inflammatory Response in a Rat Model of Acne

**DOI:** 10.1155/2023/6627393

**Published:** 2023-04-29

**Authors:** Guang Zuo, Yidan Gao, Guangtong Lu, Ming Bu, Jun Liu, Juncha Zhang, Xisheng Fan, Hao Chen, Xuesong Wang, Yanfen She

**Affiliations:** ^1^School of Acupuncture-Moxibustion and Tuina, Hebei University of Chinese Medicine, Shijiazhuang 050200, China; ^2^Department of Rehabilitation, School of Acupuncture-Moxibustion and Tuina, Hebei University of Chinese Medicine, Shijiazhuang 050200, China; ^3^Hebei International Joint Research Center for Dominant Diseases in Chinese Medicine and Acupuncture, Shijiazhuang 050091, China; ^4^Department of Experimental Acupuncture, School of Acupuncture-Moxibustion and Tuina, Hebei University of Chinese Medicine, Shijiazhuang 050200, China

## Abstract

**Background:**

The inflammatory response is an important part of the pathogenesis of acne vulgaris. Auriculotherapy has been shown to have a good therapeutic effect on this disease. The aim of this study was to explore the mechanism underlying the anti-inflammatory effect of auriculotherapy in the treatment of acne vulgaris.

**Methods:**

Propionibacterium acnes was injected subcutaneously into the ears of rats to establish an animal model of acne. The auriculotherapy intervention in rats consisted of auricular bloodletting therapy (ABT), auricular point sticking (APS), or a combination of both (ABPS). The anti-inflammatory effects of auriculotherapy were evaluated by measuring changes in ear thickness, local body surface microcirculation in the ear, and serum inflammatory factors in rats. The polarization of macrophages was analyzed by flow cytometry, and the expression of TLR2/NF-*κ*B signaling pathway in the target tissues was analyzed using western blot.

**Results:**

ABT, APS, and ABPS all reduced the erythema of ear acne, decreased microcirculation in localized ear acne, and decreased serum levels of TNF-*α* and IL-1*β* in rats. Meanwhile, the three interventions reduced M1-type macrophages and increased M2-type macrophages; only APS could reduce the expression of TLR2/NF-*κ*B signaling pathway.

**Conclusion:**

ABT, APS, and ABPS can improve the inflammatory symptoms of acne and reduce inflammatory cytokines. APS may exert anti-inflammatory effects by altering macrophage polarization and decreasing TLR2/NF-*κ*B expression.

## 1. Background

Inflammation is associated with the development of many diseases including chronic diseases and cancer. After an infection or injury, the immune system responds with a proinflammatory phase as well as an anti-inflammatory or repair phase. This is one of the characteristics of inflammation [1]. Macrophages play a crucial role in the innate immune system and the body's response to infection and can switch between M1 and M2 phenotypes [2, 3]. M1-type macrophages can be activated by stimulation of toll-like receptor ligands [2]. In the activated state, toll-like receptor ligands increase the expression, synthesis, and release of inflammatory cytokines such as tumor necrosis factor-*α* (TNF-*α*) and interleukin-1*β* (IL-*β*). M2 macrophages can be induced by interleukin-6 (IL-6) and play an important role in the elimination of infection and repair of tissue damage [4]. Therefore, tools to regulate the polarization of macrophages are important to control inflammation, and related studies have attracted great attention.

Auricular acupuncture is a traditional Chinese acupuncture treatment for inflammation, pain, or drug addiction. It stimulates various points on the outer ear (auricle). Studies have shown that auricular acupuncture has good efficacy for depression and epilepsy caused by neuroinflammation [5, 6]; it is also commonly used for dermatological diseases, inflammation of internal organs, inflammation of the otorhinolaryngologic diseases, osteoarthritis of the knee, and rheumatoid arthritis [7–9]. The anti-inflammatory effect of auriculotherapy is clear and relieves the symptoms of redness and swelling, fever, and pain from the aforementioned diseases. Some studies have shown that the therapy works through cholinergic anti-inflammatory pathways [10]. However, whether auriculotherapy can alter the polarization of macrophages has not yet been clarified.

Acne vulgaris is a chronic inflammatory skin disease of the sebaceous glands of the hair follicles. It occurs in adolescence and mainly involves the face [11]. Approximately 10% of the global population suffers from acne vulgaris, and there is a continuous increase in the number of cases [12, 13]. *P. acnes* is a Gram-positive anaerobic bacterium that is involved in the inflammatory pathogenesis of acne by binding to toll-like receptors to activate inflammatory pathways and stimulate the production of proinflammatory cytokines by keratinocytes, macrophages, and others [14].

The ear tip and the concha are commonly used sites in auricular therapy. In our preliminary experiments, we found that auricular bloodletting therapy (ABT) and/or auricular point sticking (APS) improved the redness and swelling of the ear and reduced inflammatory infiltration in an acne model rat. Therefore, this study used the same animal model and tried to explore the effect of auricular acupuncture therapy on macrophage polarization in acne disease to clarify the mechanism underlying its improvement of the inflammatory response associated with acne and to lay the foundation for further detailed studies.

## 2. Materials and Methods

### 2.1. Animals

Male Sprague-Dawley rats (*n* = 25) weighing 180-200 g (6-7 weeks old) were purchased from Beijing Vital River Laboratory Animal Technology Co., Ltd. and housed at the Experimental Animal Center of Hebei University of Chinese Medicine. All rats were acclimatized and fed for one week prior to the experiment and housed in a controlled environment (12 h light/dark cycle, 22 ± 2°*C*, and 55 ± 5% relative humidity) with free access to food and water. The environment and animals in this study were SPF grade and were never used for other research procedures. All experimental procedures were performed in strict accordance with the Guidance Suggestions for the Care and Use of Laboratory Animals (developed by the Ministry of Science and Technology, China). The study protocol was obtained from the Experimental Animal Management and Ethics Committee of Hebei University of Chinese Medicine (license number: DWLL202203131), and all animal handling procedures were performed in accordance with the Guidelines for the Protection and Use of Laboratory Animals of Hebei University of Chinese Medicine.

### 2.2. Experimental Grouping

After 1 week of acclimatization, the 25 SD rats were randomly divided into five groups: blank control group (control), acne model group (acne), auricular bloodletting therapy (ABT), auricular point sticking (APS), and auricular bloodletting therapy combined with auricular point sticking (ABPS).

### 2.3. Interventions

**Figure 1:**
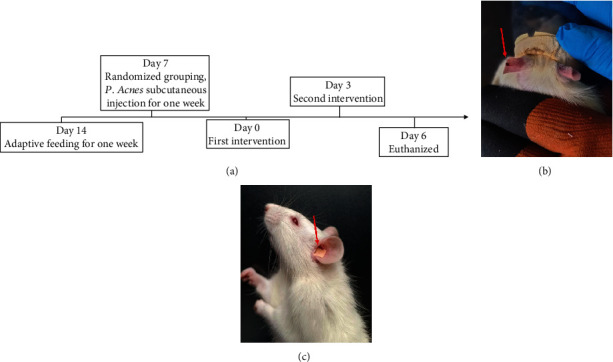
Experimental roadmap and interventions. (a) The roadmap of this experiment. (b) The schematic diagram of the operation of auricular bloodletting therapy (ABT). (c) The schematic diagram of the operation of auricular point sticking (APS).

Rats in the blank group (*n* = 5) were injected subcutaneously with 0.9% saline in the right ear. The remaining four groups of rats (*n* = 20) were modeled by subcutaneous injection of *P. acnes* (3 × 10^9^ cfu/ml, 20 *μ*l/each, Shanghai Yan Sheng) in the right ear for seven consecutive days [15]. Rats were fixed by rat fixators when they were intervened. ABT was performed by pricking the ear tip using a sterile 1 ml syringe using the left ear contralateral point as the stimulation point. The site was subject to bleeding (five drops). This was repeated two times a week for one week of intervention. In the APS group, the auricular nail patch was applied to the left auricular concha with a pressure of about 2-5 ibf (WAGNER, USA) for two minutes in the morning, two minutes at noon, and two minutes in the evening; the auricular nail patch was removed after the pressure was applied. The ABPS group performed ABT first followed by APS. The specific schedule and interventions of the experiment are shown in Figure 1.

### 2.4. General Observation of the Rat

The redness and swelling of the rats' ears were monitored throughout the experimental period. In detail, on day 6, we used an electronic vernier caliper (AIRAJ, Germany, arz-1331) on the right ear of the rat to measure the thickness of acne. This measurement procedure would be repeated 3 times, and then, its average value would be calculated. Finally, the local thickness of the acne was obtained. Similarly, we photographed and preserved localized acne in the ears of rats.

### 2.5. Histological Analysis

We used 20% uratan (0.7 ml/100 g) to anesthetize the rats on day 6. The right ear was taken and placed in 4% paraformaldehyde and fixed at room temperature for at least 24 hours. After fixation, it was paraffin-embedded, sectioned (5 *μ*m), dewaxed, rehydrated, and stained with hematoxylin and eosin (HE). Histopathological changes were examined by light microscopy with any range of magnification for each microscopic field of view at 40x magnification.

### 2.6. Body Surface Microcirculation of Ear

Rats are placed on the bench after 20% uratan anesthesia on day 6. The laser scatter flow imager (moor FLPI-2, UK) was turned on 20 minutes in advance to stabilize the machine performance. During the test, the scanning lens of the instrument was pointed at the head of the rat, and the instrument was automatically focused. Each rat was probed for two minutes. The monitored data were processed using the moorFLPI-2 Review V50 software. The right ear of the implanted *P. acnes* was defined as the region of interest, and the mean perfusion volume in units of PU (perfusion unit) was recorded for a 25 mm^2^ circular area within the region of interest.

### 2.7. Flow Cytometry (FCM)

Rats were euthanized by overanesthesia, disinfected with 75% alcohol on the body surface, and the entire spleen was removed by dissection. The spleen was then washed with ImunoSep cell sorting solution (PBM, China), minced, and placed in a 50 ml test tube with a stainless-steel screen. A single cell suspension was prepared by grinding the spleen with a 5 ml rubber tip syringe while adding ImunoSep cell sorting solution dropwise. Erythrocyte lysis was performed using erythrocyte lysis solution (PBM, China). The cell suspensions were incubated with CD68-PE-Vio770 (Miltenyi, Germany), CD86-PE (Thermo Fisher, USA), and CD163-FITC (Bio-Rad, USA) for 30 min at room temperature followed by flow cytometry (ThermoFisher Attune NxT, USA) for detection. The experimental data were analyzed by Attune NxT software.

### 2.8. Cytokine Measurements

After the rats were anesthetized, we used abdominal aortic blood sampling to obtain blood. The blood samples were left to stand at room temperature for 3 h. The blood was then centrifuged at 4°C for 15 min at 3000 × g. Finally, the serum was taken from the upper layer of the centrifuge tube. The concentrations of TNF-*α* and IL-1*β* in serum were analyzed with rat-specific enzyme-linked immunosorbent assay (ELISA) kits (Beyotime, China) according to the manufacturer's instructions.

### 2.9. Western Blot

The tissue used was the right ear of the rat, which is where the acne model is located. The tissues of each subgroup were taken separately, washed with cold PBS, and homogenized in a lysis solution (Bestbio, China) supplemented with protease inhibitors and phosphatase inhibitors. The tissue lysis products were centrifuged at 12,000 × g for 10 min at 4°C, and the supernatant was obtained. The concentration was determined using the BCA method. Equal amounts of proteins were separated by polypropylene phthalamide gel electrophoresis (SDS-PAGE) and then transferred onto the polyvinylidene difluoride (PVDF) membrane. The PVDF membrane was blocked in TBST buffer containing 5% BSA for 3 h at room temperature, and then, specific antibodies were added and left overnight at 4°C. PVDF membranes were washed five times with TBST at room temperature and incubated with a secondary antibody at room temperature for 1 h. PVDF membranes were rinsed again with TBST five times. The ECL kit (Sharebio, China) chemofluorescence method was used to cover the strips, and a fully automated exposure machine was used for exposure (GelView 6000Plus, China). Band densities were quantified using ImageJ software (version 1.52a, National Institutes of Health, Bethesda, MD, USA). In this experiment, primary antibodies were used including NF-*κ*B p65 (Cell Signaling Technology, USA), TLR2 (Abcam, UK), p-NF-*κ*B p65 (Cell Signaling Technology, USA), *β*-actin (Abways, China), and HRP (Abways, China).

### 2.10. Statistical Analysis

SPSS 21.0 statistical software (IBM SPSS Statistics, SPSS Inc., Chicago, IL, USA) was used to statistically analyze all experimental data, and the measures were expressed as mean ± standard deviation; the count data were expressed as percentages. One-way analysis of variance (ANOVA) was used for comparison between groups. The LSD method test was used with a significance level of *P* < 0.05.

## 3. Results

### 3.1. ABT, APS, and ABPS Suppressed *P. acnes*-Mediated Skin Inflammation in Rats

**Figure 2:**
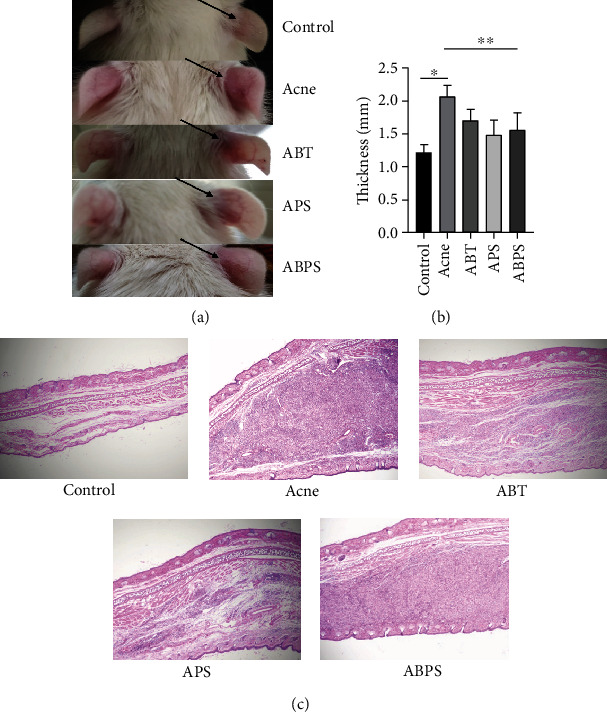
ABT, APS, and ABPS inhibit skin inflammation induced by *P. acnes* in rats. (a) Representative images of rats taken on day 6. (b) Comparison of the thickness of localized acne in the ears of rats on day 6. (c) Representative images of HE staining of localized acne in rat ears. The values represent the mean ± standard deviation of five independent experiments. *N* = 5, ^∗^ indicates *P* < 0.05, and ^∗∗^ indicates *P* < 0.05.

We first evaluated the anti-inflammatory effects of ABT, APS, and ABPS on acne model rats. Histological observations of the injection site showed that *P. acnes* caused a significant increase in the infiltration of inflammatory cells into the site. Clearly, the injection of 0.9% saline alone did not cause the ears of rats to become swollen (Figures 2(a) and 2(c)). Histological images also showed that ABT, APS, and ABPS all reduced the development of granulomatous changes in response to *P. acnes* exposure compared to the acne group (Figure 2(c)). To evaluate the inflammatory changes more objectively and accurately, we measured the changes in the thickness of localized acne in the ears of acne model rats, and the three interventions reduced the thickness compared to the model group (*P* < 0.05, Figure 2(b)).

### 3.2. Localized Changes in Body Surface Microcirculation in Acne Vulgaris in Rats

**Figure 3:**
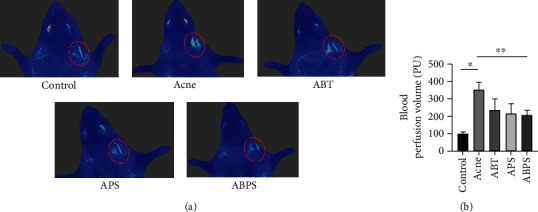
Localized changes in body surface microcirculation in acne vulgaris in rats. (a) Representative images of localized body surface microcirculation in acne. (b) Statistical results of body surface microcirculation. The values represent the mean ± standard deviation of five independent experiments. *N* = 5, ^∗^ indicates *P* < 0.05, and ^∗∗^ indicates *P* < 0.05.

We did body surface imaging on acne model rats. In other words, we also measured the localized body surface microcirculation in acne, and the local blood flow was significantly increased in the model group (*P* < 0.05, Figure 3(a)). Compared to the model group, ABT, APS, and ABPS all reversed this alteration (*P* < 0.05, Figure 3(b)).

### 3.3. ABT, APS, and ABPS Can Regulate Macrophage Polarization

**Figure 4:**
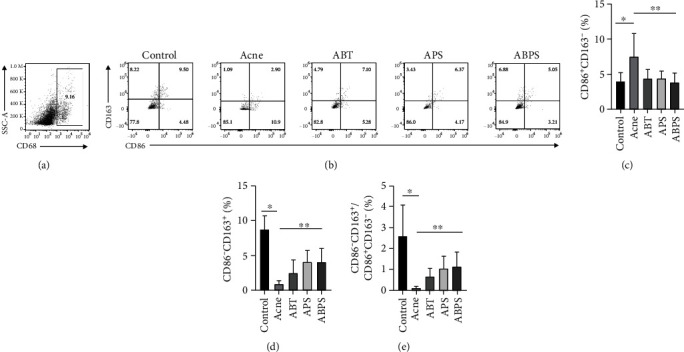
ABT, APS, and ABPS can regulate macrophage polarization. (a, b) The flow cytometry sorting of M1/M2 positive cells from splenic macrophages with CD68^+^-labeled total macrophages, CD68^+^CD86^+^ CD163^−^-labeled M1 macrophages, and CD68^+^CD86^−^CD163^+^-labeled M2 macrophages. (c) The percentage of M1-type macrophages in each group. (d) The percentage of M2-type macrophages in each group. (e) The ratio of M2/M1 in each group. The values represent the mean ± standard deviation of four independent experiments. *N* = 4, ^∗^ indicates *P* < 0.05, and ^∗∗^ indicates *P* < 0.05.

ABT, APS, and ABPS improved the inflammatory manifestations of acne such as redness and swelling. To explore the mechanism, we examined macrophage polarization in the spleen of rats. Subcutaneous injection of *P. acnes* into rat ears increased the proportion of splenic M1-type macrophages in the model group compared with the blank group (*P* < 0.05, Figure 4(b)). Versus the model group, ABT, APS, and ABPS could all reduce the proportion of M1-type macrophages and induce the conversion of M2-type macrophages (*P* < 0.05). No differences were shown between the three interventions (*P* > 0.05, Figures 4(c)–4(e)).

### 3.4. ABT, APS, and ABPS Decreased the Expression of TNF-*α*, IL-1*β*, and TLR2/NF-*κ*B Signaling Pathways

**Figure 5:**
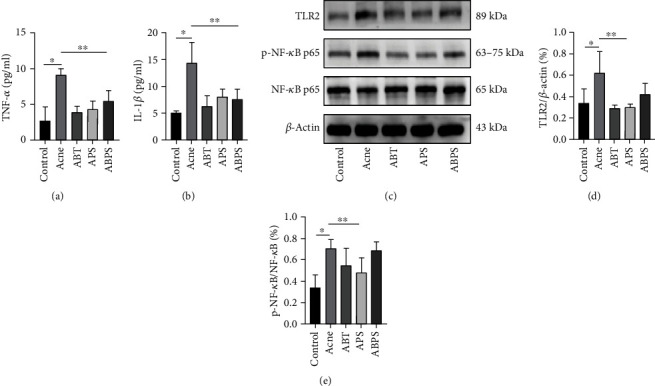
ABT, APS, and ABPS decreased the expression of TNF-*α*, IL-1*β*, and TLR2/NF-*κ*B signaling pathways. (a, b) Serum TNF-*α* and IL-1*β* levels. (c–e) Western blot analysis of TLR2, p-NF-*κ*B p65, and NF-*κ*B p65. The values represent the mean ± standard deviation of three independent experiments. *N* = 3, ^∗^ indicates *P* < 0.05, and ^∗∗^ indicates *P* < 0.05.

We also measured the levels of TNF-*α* and IL-1*β* in the serum of rats. Figures 5(a) and 5(b) show that the inflammatory factors in the serum of the model group were significantly increased versus the blank group (*P* < 0.05). ABT, APS, and ABPS decreased the secretion of inflammatory factors relative to the model group (*P* < 0.05). Again, no differences were shown between the three interventions (*P* > 0.05). However, western blot results showed that only APS decreased TLR2/NF-*κ*B expression (*P* < 0.05). ABT and ABPS did not alter NF-*κ*B phosphorylation levels (*P* > 0.05, Figures 5(c)–5(e)).

## 4. Discussion

Acne vulgaris (AV) is a chronic inflammatory disease [16]. *Propionibacterium acnes* plays an important role in the pathogenesis of acne [17]. Therefore, in the present study, we used a *P. acnes* bacterial solution injected subcutaneously into the right ear of rats to induce acne. This is a recognized inflammatory model of acne [15]. The results also showed localized acne-like manifestations in the ear of rats such as reddening welts, swelling, and purulent discharge. We selected representative ABT and APS from our previous clinical studies with the aim of exploring their anti-inflammatory effects on acne [18, 19]. The preliminary clarification of the mechanism of this therapy will lay the foundation for the next in-depth study of auriculotherapy.

Our results showed that ABT, APS, and ABPS can improve the inflammatory manifestations of acne. The moorFLPI-2 laser scatter flow imaging system can record the mean blood perfusion at the acne site [20]. When combined with the measured acne thickness, these can assess the inflammatory changes in acne. Thus, ABT and APS can reduce inflammatory infiltration at the acne site and reduce exudation, which can help reverse inflammatory deterioration. However, a between-group comparison of ABT, APS, and ABPS showed that the combination of ABT and APS did not enhance the anti-inflammatory effect, which is not consistent with our previous clinical study [19]. This may be because ABT pierces the skin, and rats scratch their ears or tear each other, which may reduce the efficacy of this intervention.

The spleen is the largest secondary lymphoid organ in the body. The location of immune cells dispersed in the spleen, and their ability to migrate out of the spleen gives the spleen an important role in the immune system [21]. Regarding the choice of markers for splenic macrophages, this work refers to the studies of Crum et al. and Rubio-Navarro et al. [22, 23] using CD68^+^ to label total macrophages in the spleen, CD68^+^ CD86^+^ CD163^−^ to denote M1-type macrophages, and CD68^+^ CD86^−^ CD163^+^ to denote M2-type macrophages.

Studies have shown that acupuncture of the auricular concha area activates the vagal cholinergic anti-inflammatory pathway [10], but the mechanism of action of ABT is unclear. Tanaka et al. and McAllen et al. [24, 25] found that sympathetic nerves are involved in the immune regulation of inflammation. Specifically, the organism synthesizes and secretes complement components or cytokines—including more sensitive ones such as TNF-*α* and IL-1*β*—when systemic or local infections occur [26]. These inflammatory factors influence the immune system. The splanchnic anti-inflammatory pathway is thought to be a neuroimmune reflex and can regulate the inflammatory response that influences the immune system by controlling immune function through the greater splanchnic nerves [27]. More importantly, Katayama demonstrated that vagus nerve stimulation can activate the vagosympathetic reflex mediated by the splenic nerve, thus altering the splenocyte phenotype for anti-inflammatory protective effects. These studies demonstrate a strong correlation between the vegetative nervous system, the spleen, and anti-inflammation. Similarly, our experiment found that auriculotherapy altered splenic macrophage polarization. This may be the mechanism by which auriculotherapy exerts its anti-inflammatory effects.

A dysfunctional spleen increases the risk of serious infections [28]. Large numbers of mononuclear macrophages are present in the subcapsular red pulp of the spleen and leave the spleen during inflammation and move to the inflamed tissue [29]. Upon reaching the diseased tissue, as in acne, these mononuclear macrophages release large amounts of inflammatory cytokines and promote the recruitment of neutrophils—this process promotes inflammation while also prompting tissue repair but also potentially causes more severe inflammation [30]. This was corroborated by our flow cytometry results where CD86^+^ CD163^−^ (M1-type) cells were present in the macrophages of the spleen of rats in the blank group, and CD86^−^ CD163^+^ (M2-type) cells were kept in balance. In contrast, rats in the model group injected with *P. acnes* had an increased proportion of M1-type cells and a decreased proportion of M2-type cells. Severe inflammation occurred locally to the injection. Interestingly, ABT, APS, and ABPS rebalanced the ratio of M2/M1 macrophages and reduced the local inflammatory infiltration of the tissue.

Macrophage polarization plays an important role in the development of many other diseases. Acupuncture can regulate cell signaling pathways and cytokine expression and secretion, which can modulate macrophage polarization, thus resulting in anti-inflammatory effects [31]. For example, Tian et al. [32] found that electroacupuncture could regulate macrophage polarization in the stomach of rats in a diabetic model to protect Cajal mesenchymal cells. Wang et al. [33] showed that electroacupuncture could reduce the concentration of proinflammatory mediators in vivo and regulate the polarization of adipose tissue macrophages, thus preventing obesity. In studies related to acne vulgaris, few studies have mentioned changes in macrophages. This work provides preliminary evidence of the anti-inflammatory effects of ABT and APS in relation to macrophage polarization. It is unclear about how the polarization of splenic macrophages is regulated, and further experiments are needed for an in-depth study.

To address the pathogenesis of acne vulgaris, we examined the expression of TNF-*α*, IL-1*β* in serum, and TLR2/NF-*κ*B signaling pathway in tissues. It was shown that H19 signaling in keratin-forming cells exacerbates the inflammatory response through the miR-196A/TLR2/NF-*κ*B pathway [34, 35]. Specifically, peptides from *P. acnes* can bind to TLR2 on the membranes of keratinocytes and macrophages and then phosphorylate NF-*κ*B, thus promoting the secretion of TNF-*α*, IL-1*β*, etc. These proinflammatory factors are responsible for the worsening of inflammation after the onset of acne resulting in hyperkeratosis with severe swelling, inflammatory infiltration, and granulomatous reaction [36]. As shown in Figures 5(a) and 5(b), ABT, APS, and ABPS all reduce the secretion levels of TNF-*α* and IL-1*β*. Interestingly, ABT could not inhibit the phosphorylation of NF-*κ*B, although it could reduce the expression of TLR2. Similarly, the combination of the two did not show better effects; ABT also seems to counteract some of the effects of APS. This suggests that ABT exerts an anti-inflammatory and may act on other signaling pathways; further studies are thus needed. Nevertheless, APS nicely inhibited the TLR2/NF-*κ*B signaling pathway.

However, there are several limitations in the present study. *P. acnes* is probably not the only inflammatory stimulus. Keratin and various lipid products are important inflammatory agents. The proportion of localized macrophage polarization in acne is unknown. In this study, we did not conduct an additional intervention on the control group or acne group, which generated some bias in the model. In the supplementary material (available here). We added a nonactive point group to reduce experimental bias. Moreover, further research is warranted.

## 5. Conclusion

In summary, ABT, APS, and ABPS have anti-inflammatory effects in acne model rats. This may be related to macrophage polarization. APS may improve acne inflammation by inhibiting TLR2/NF-*κ*B signaling pathway, while ABT does not inhibit NF-*κ*B phosphorylation. This study provides a scientific basis for auricular acupuncture therapy for the treatment of acne vulgaris and lays the foundation for more in-depth studies on the anti-inflammatory effects exerted by auriculotherapy in the future.

## Data Availability

The data used to support the findings of this study are available from the corresponding author on request.

## Supplementary Materials

Supplementary Materials In order to reduce bias, we have added a sham group treated with tail bloodletting and tail point sticking therapy. The results of the skin of the ear, thickness of localized acne, and body surface microcirculation in acne are shown in Figure S1. Figure S1: (a) representative images of rats taken on day 6. (b) Comparison of the thickness of localized acne in the ears of rats on day 6. (c) Representative images of localized body surface microcirculation in acne. (d) Statistical results of body surface microcirculation. *N* = 5, ^∗^ indicates *P* < 0.05, and ^∗∗^ indicates *P* < 0.05. *P. acnes* relative to 0.9% saline could cause the ears of rats to become red, swollen, and rough (*P* < 0.05, Figure S1a). ABT, APS, and ABPS could all reduce these symptoms (*P* < 0.05, Figure S1a). However, in nonactive point, the sham group did not have similar anti-inflammatory effects (*P* < 0.05, Figure S1). To evaluate the inflammatory changes more objectively and accurately, we measured the changes in the thickness of localized acne in the ears of acne model rats, and the three interventions reduced the thickness compared to the acne and sham group (Figure S1b).

## References

[1] Vannella K. M., Wynn T. A. (2017). Mechanisms of organ injury and repair by macrophages. *Annual Review of Physiology*.

[2] Zhang L., Wang C. C. (2014). Inflammatory response of macrophages in infection. *Hepatobiliary & Pancreatic Diseases International*.

[3] Chinetti-Gbaguidi G., Staels B. (2011). Macrophage polarization in metabolic disorders: functions and regulation. *Current Opinion in Lipidology*.

[4] Gordon S., Martinez F. O. (2010). Alternative activation of macrophages: mechanism and functions. *Immunity*.

[5] Lei W. (2016). *Comparative Study on the Localization and Diagnosis and Treatment Procedures in Two Ear Acupuncture Systems in China and Europe*.

[6] Wu J. Summary and analysis of superior diseases of auricular acupuncture therapy.

[7] Pavlov V. A., Chavan S. S., Tracey K. J. (2018). Molecular and functional neuroscience in immunity. *Annual Review of Immunology*.

[8] Koopman F. A., Chavan S. S., Miljko S. (2016). Vagus nerve stimulation inhibits cytokine production and attenuates disease severity in rheumatoid arthritis. *Proceedings of the National Academy of Sciences of the United States of America*.

[9] Bonaz B., Sinniger V., Hoffmann D. (2016). Chronic vagus nerve stimulation in Crohn's disease: a 6-month follow-up pilot study. *Neurogastroenterology and Motility*.

[10] Ouyang X., Li S., Zhou J., Chen J. D. (2020). Electroacupuncture ameliorates gastric hypersensitivity via adrenergic pathway in a rat model of functional dyspepsia. *Neuromodulation*.

[11] Qiang J. (2019). Chinese acne treatment guidelines. *Journal of Clinical Dermatology*.

[12] Tan J. K., Bhate K. (2015). A global perspective on the epidemiology of acne. *British Journal of Dermatology*.

[13] Shunqiao J., Xicong Z., Weiqing Z., Yuxue G. (2018). Clinical observation on the treatment of moderate and severe acne with intense pulsed light combined with acid. *Chinese Journal of Dermatology and Venereology*.

[14] Huang W. C., Tsai T. H., Chuang L. T., Li Y. Y., Zouboulis C. C., Tsai P. J. (2014). Anti-bacterial and anti-inflammatory properties of capric acid against *Propionibacterium acnes*: a comparative study with lauric acid. *Journal of Dermatological Science*.

[15] De Young L. M., Young J. M., Ballaron S. J., Spires D. A., Puhvel S. M. (1984). Intradermal injection of *Propionibacterium acnes*: a model of inflammation relevant to acne. *Journal of Investigative Dermatology*.

[16] Dreno B. (2017). What is new in the pathophysiology of acne, an overview. *Journal of the European Academy of Dermatology and Venereology*.

[17] Fernández J. R., Webb C., Rouzard K. (2018). SIG1459: a novel phytyl-cysteine derived TLR2 modulator with in vitro and clinical anti-acne activity. *Experimental Dermatology*.

[18] Yayu G., Man Z., Yanfen S. (2022). Observation on the efficacy of auricular point pricking at different frequencies combined with auricular point pressing in the treatment of acne vulgaris. *Chinese Journal of Acupuncture and Moxibustion*.

[19] Song Y.-j., Fan X.-s., Li M.-y. (2019). Treatment of acne vulgaris with auricular acupoint pricking-bloodletting plus auricular point sticking therapy: a randomized controlled study. *Journal of Acupuncture and Tuina Science*.

[20] Vaz P. G., Humeau-Heurtier A., Figueiras E., Correia C., Cardoso J. (2016). Laser speckle imaging to monitor microvascular blood flow: a review. *IEEE Reviews in Biomedical Engineering*.

[21] Lewis S. M., Williams A., Eisenbarth S. C. (2019). Structure and function of the immune system in the spleen. *Science Immunology*.

[22] Crum R. J., Hall K., Molina C. P. (2022). Immunomodulatory matrix-bound nanovesicles mitigate acute and chronic pristane-induced rheumatoid arthritis. *Regenerative Medicine*.

[23] Rubio-Navarro A., Guerrero-Hue M., Martín-Fernandez B. (2016). Phenotypic characterization of macrophages from rat kidney by flow cytometry. *J. Vis. Exp.*.

[24] Tanaka S., Abe C., Abbott S. (2021). Vagus nerve stimulation activates two distinct neuroimmune circuits converging in the spleen to protect mice from kidney injury. *Proceedings of the National Academy of Sciences of the United States of America*.

[25] McAllen R. M., McKinley M. J., Martelli D. (2022). Reflex regulation of systemic inflammation by the autonomic nervous system. *Autonomic Neuroscience-Basic & Clinical*.

[26] Bronte V., Pittet M. J. (2013). The spleen in local and systemic regulation of immunity. *Immunity*.

[27] Martelli D., Farmer D. G., Yao S. T. (2016). The splanchnic anti-inflammatory pathway: could it be the efferent arm of the inflammatory reflex?. *Experimental Physiology*.

[28] Edgren G., Almqvist R., Hartman M., Utter G. H. (2014). Splenectomy and the risk of sepsis. *Annals of Surgery*.

[29] Swirski F. K., Nahrendorf M., Etzrodt M. (2009). Identification of splenic reservoir monocytes and their deployment to inflammatory sites. *Science*.

[30] Mu X., Li Y., Fan G. C. (2021). Tissue-resident macrophages in the control of infection and resolution of inflammation. *Shock*.

[31] Wang J., Lu S., Yang F. (2021). The role of macrophage polarization and associated mechanisms in regulating the anti-inflammatory action of acupuncture: a literature review and perspectives. *Chinese Medicine*.

[32] Tian L., Song S., Zhu B., Liu S. (2018). Electroacupuncture at ST-36 protects interstitial cells of Cajal via sustaining heme oxygenase-1 positive M2 macrophages in the stomach of diabetic mice. *Oxidative Medicine and Cellular Longevity*.

[33] Wang H. F., Chen L., Xie Y. (2021). Electroacupuncture facilitates M2 macrophage polarization and its potential role in the regulation of inflammatory response. *Biomedicine & Pharmacotherapy*.

[34] Yang S., Fang F., Yu X. (2020). Knockdown of H19 inhibits the pathogenesis of acne vulgaris by targeting the miR-196a/TLR2/NF-*κ*B axis. *Inflammation*.

[35] Lee H., Hwang D., Lee M. (2022). Micro-current stimulation suppresses inflammatory responses in peptidoglycan-treated raw 264.7 macrophages and Propionibacterium acnes-induced skin inflammation via TLR2/NF-*κ*B signaling pathway. *International Journal of Molecular Sciences*.

[36] Kurokawa I., Danby F. W., Ju Q. (2009). New developments in our understanding of acne pathogenesis and treatment. *Experimental Dermatology*.

