# Supplementary figures and images for: Auriculotherapy Modulates Macrophage Polarization to Reduce Inflammatory Response in a Rat Model of Acne
Source: Mediators Inflamm. 2023 Apr 29;2023:6627393. doi: 10.1155/2023/6627393 (PMC10163966; doi:10.1155/2023/6627393)

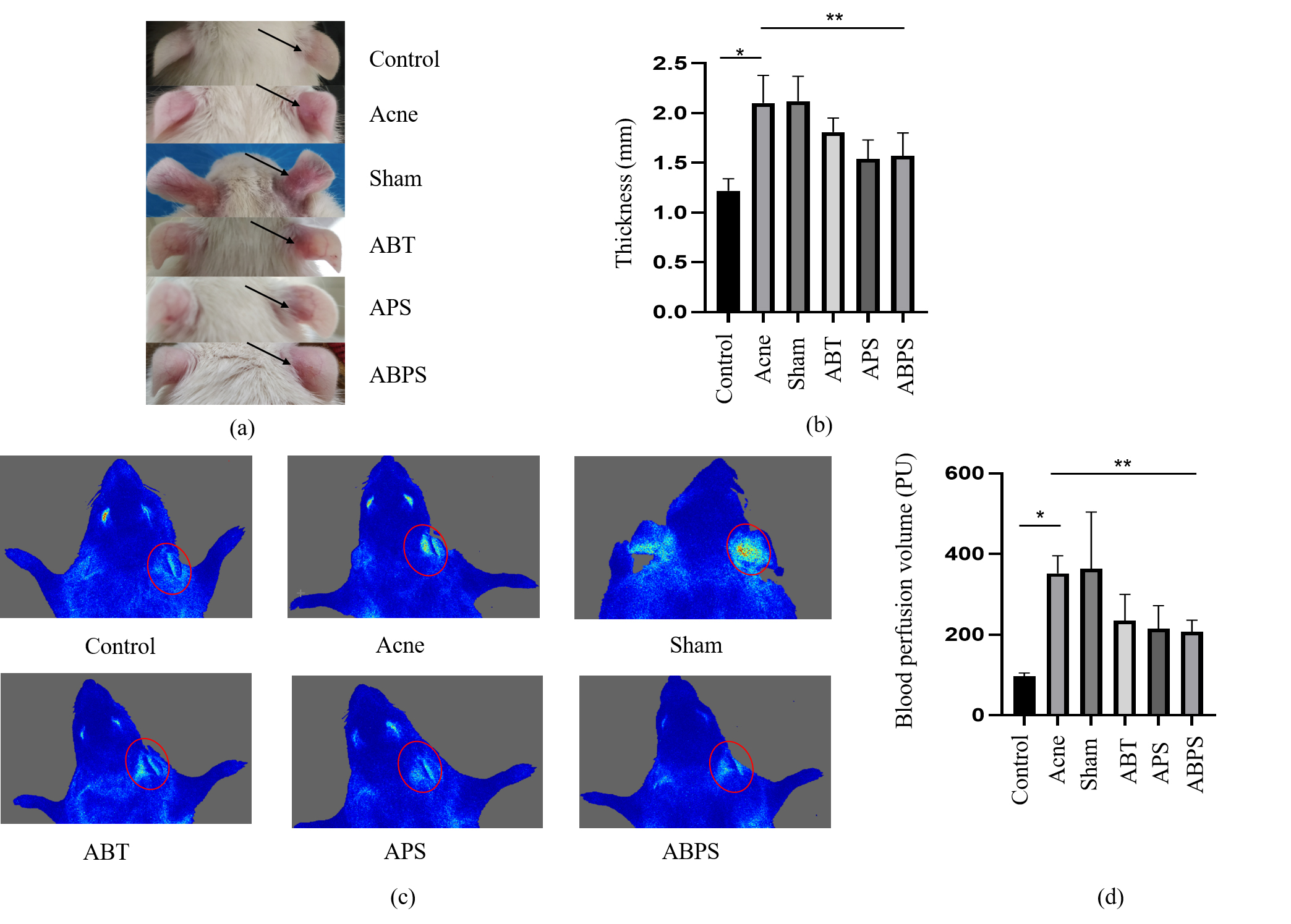

Supplement: Supplementary Materials — In order to reduce bias, we have added a sham group treated with tail bloodletting and tail point sticking therapy. The results of the skin of the ear, thickness of localized acne, and body surface microcirculation in acne are shown in Figure S1. Figure S1: (a) representative images of rats taken on day 6. (b) Comparison of the thickness of localized acne in the ears of rats on day 6. (c) Representative images of localized body surface microcirculation in acne. (d) Statistical results of body surface microcirculation. N = 5, ∗ indicates P < 0.05, and ∗∗ indicates P < 0.05. P. acnes relative to 0.9% saline could cause the ears of rats to become red, swollen, and rough (P < 0.05, Figure S1a). ABT, APS, and ABPS could all reduce these symptoms (P < 0.05, Figure S1a). However, in nonactive point, the sham group did not have similar anti-inflammatory effects (P < 0.05, Figure S1). To evaluate the inflammatory changes more objectively and accurately, we measured the changes in the thickness of localized acne in the ears of acne model rats, and the three interventions reduced the thickness compared to the acne and sham group (Figure S1b). [file 6627393.f1.jpg]
